# *TCF2* Attenuates FFA-Induced Damage in Islet β-Cells by Regulating Production of Insulin and ROS

**DOI:** 10.3390/ijms150813317

**Published:** 2014-07-30

**Authors:** Xiaojuan Quan, Lin Zhang, Yingna Li, Chunlian Liang

**Affiliations:** Department of Geriatrics, the Second Affiliated Hospital, Medical School of Xi’an Jiaotong University, Xi’an 710004, China; E-Mails: mengjingcn@mail.xjtu.edu.cn (L.Z.); wxd.wxd@mail.xjtu.edu.cn (Y.L.)

**Keywords:** β-cells, free fatty acids, TCF2, insulin, reactive oxygen species

## Abstract

Free fatty acids (FFAs) are cytotoxic to pancreatic islet β-cells and play a crucial role in the diabetes disease process. A recent study revealed a down-regulation of transcription factor 2 (TCF2) levels during FFA-mediated cytotoxicity in pancreatic β-cells. However, its function during this process and the underlying mechanism remains unclear. In this study, treatment with palmitic acid (PA) at high levels (400 and 800 μM) decreased β-cell viability and TCF2 protein expression, along with the glucose-stimulated insulin secretion (GSIS). Western and RT-PCR analysis confirmed the positive regulatory effect of *TCF2* on GSIS through promotion of the key regulators pancreatic duodenal homeobox-1 (PDX1) and glucose transporter 2 (GLUT2) in β-cells. In addition, both PI3K/AKT and MEK/ERK showed decreased expression in PA (800 μM)-treated β-cells. Overexpression of *TCF2* could effectively restore the inhibitory effect of PA on the activation of PI3K/AKT and MEK/ERK as well as β-cell viability, simultaneously, inhibited PA-induced reactive oxygen species (ROS) generation. After blocking the PI3K/AKT and MAPK/ERK signals with their specific inhibitor, the effect of overexpressed TCF2 on β-cell viability and ROS production was obviously attenuated. Furthermore, a protective effect of TCF2 on GSIS by positive modulation of JNK-PDX1/GLUT2 signaling was also confirmed. Accordingly, our study has confirmed that *TCF2* positively modulates insulin secretion and further inhibits ROS generation via the PI3K/AKT and MEK/ERK signaling pathways. Our work may provide a new therapeutic target to achieve prevention and treatment of diabetes.

## 1. Introduction

The rapid increase of diabetes mellitus (DM) worldwide has become a serious threat to human health. Although the research on treatment and risk factors (obesity, genetic susceptibility, and mental pressure) of DM has achieved great progress, the population of DM patients is still increasing rapidly [1]. What is even more frustrating is that the causes and the signaling pathways involved in the regulation of abnormal glycolipid metabolism and insulin resistance (IR) are still unknown.

Pancreatic β-cells play a key role in insulin secretion-related glucose homeostasis [2]. The viability and apoptosis of β-cells is dynamically involved in the pathogenesis of diabetes and ranks as a main cause of diabetes [3]. Despite mechanistic differences, the dysfunction of pancreatic β-cells is a common feature of both type 1 and late-stage type 2 diabetes [4,5]. Thereforem, to explore the underlying mechanism will contribute to understanding of the pathogenesis of diabetes, which is critical for prevention and control of diabetes.

IR represents a hallmark of type 2 DM caused by disturbances of glucose and lipid metabolism associated with obesity [6]. The inability of β-cells to decrease insulin secretion is sufficient to compensate for IR and will lead to type 2 DM. Hence, the decreased insulin secretion in pancreatic β-cells is another leading cause of type 2 DM. Research has confirmed that the dysfunction of the β-cell is one of major characteristics in the pathogenesis of type 2 diabetes, and can be caused by some factors, such as continuing high levels of blood glucose and free fatty acids (FFAs).

FFAs are molecules released from lipids and enzyme action that possess diverse and confirmed to be associated with the process of IR. Recent research found that the elevated plasma FFA levels in the obese resulted from enlarged and stressed adipose tissue release and reduced FFA clearance. Palmitic acid (PA) is one of the most common FFAs found in animals. Studies indicate that elevated circulating FFAs may directly contribute to the development of type 2 DM [7] as its lipotoxic for islet β-cells [8]. Furthermore, evidence from *in vivo* studies shows that elevated FFAs represent a crucial link to the onset of IR and β-cell dysfunction, that even result in apoptosis, initiation of diabetes, and promotion of disease progression [8,9]. However, the precise molecular mechanism involved in this progress remains unclear.

Transcription factor 2 (TCF2) plays a crucial role in the specific regulation of gene expression in pancreatic islets and many other tissues [10,11]. A large amount of maturity-onset diabetes of the young, type 5 is caused by TCF2 mutations, indicating a significant correlation between TCF2 and diabetes [12,13]. It has been proven that the mutation of *TCF2* in the liver causes a syndrome of pancreatic exocrine dysfunction and glucose intolerance, impairs insulin signaling, and promotes hepatic gluconeogenesis and diabetes [14]. Besides, it has also been confirmed that down-regulation of *TCF2* gene expression accompanies PA-mediated cytotoxicity in cultured pancreatic islet β-cells [15], and the specific regulatory effect of FFAs on TCF2 and its molecular mechanism remain unknown.

A recent study revealed that TCF2 expression was down-regulated upon the elevation of plasma FFA levels [16]. Therefore, we aimed to explore the effect of FFAs on pancreatic islet β-cells and the role of *TCF2* in this process. In this study, to determine the lipotoxicity of FFAs in β-cells, we pretreated islet β-cells INS-1 *in vitro* with the increasing concentrations of PA. Furthermore, the underlying molecular mechanism of FFAs lipotoxicity to islet β-cells was also investigated.

## 2. Results

### 2.1. Transcription Factor 2 (TCF2) Relieved Free Fatty Acids (FFA) Induced Inhibitory Effect on INS-1 Cell Viability

**Figure 1:**
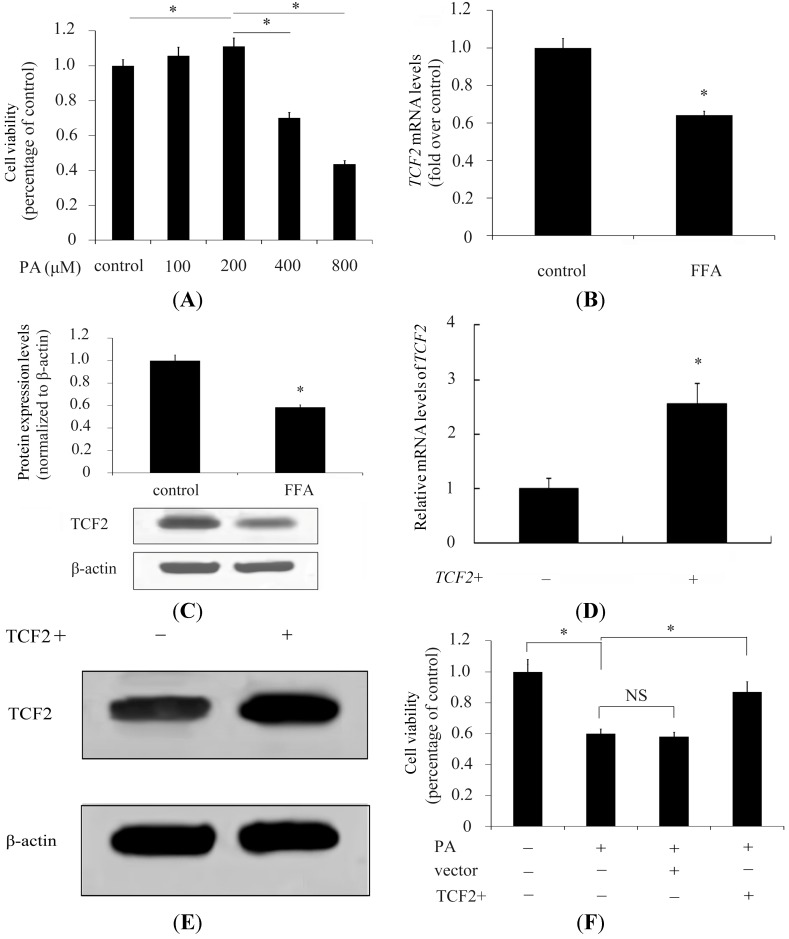
High concentrations of palmitic acid (PA) inhibit the expression of *transcription factor 2* (*TCF2*) in β-cells. Viability of INS-1 β-cells was analyzed after treatment with a concentration gradient concentration of PA for 48 h, then 0.5 mg/mL 3-(4,5-dimethyl-2-thiazolyl)-2,5-diphenyl-2*H*-tetrazolium bromide (MTT) was added and cells were cultured for further 4 h to evaluate cell viability (**A**); *TCF2* expression was evaluated by RT-PCR (**B**); and Western blotting (**C**) in INS-1 cells with or without (control) pretreatment with PA (800 μM); The transfection effect of adenovirus containing the TCF2 was evaluated by RT-PCR (**D**); And Western blotting (**E**); Viability of INS-1 cells pretreated with PA (800 μM) and overexpressing *TCF2* or transfected with the control vector was analyzed (**F**). * *p* < 0.05. NS means no statistically significant.

It is widely known that FFAs are implicated in obesity, IR, and DM. A previous study confirmed that increased levels of FFAs were positively correlated with the deterioration of β-cell function [7,17]. We initially examined the effect of FFA treatment on cell viability in INS-1 pancreatic β-cells. The results showed that at a certain range of PA concentration (lower than 200 μM), the viability of INS-1 cells showed a dose-dependent decrease (Figure 1A). However, a dramatically decrease in cell viability was detected when the PA concentration exceeded 400 μM (71% increase over control) and viability was even lower with 800 μM PA (43% increase over control; Figure 1A). These results confirmed the toxic effect of high concentrations of FFAs on β-cells. *TCF2* encodes the protein Hnf1b, which was decreased in INS-1 pancreatic β-cells pretreated with PA [15]. *TCF2* expression was further analyzed using RT-PCR and Western blotting; the results showed a significant decrease of TCF2 mRNA level concomitant with a decrease of *TCF2* protein in β-cells pretreated with FFAs (Figure 1B,C). To further evaluate the role of *TCF2* in FFA-induced cell viability, INS-1 cells were transfected with recombinant adenovirus vectors to overexpress *TCF2*. The results showed that transfection with recombinant adenovirus vectors significantly induced *TCF2* mRNA and protein expression levels in INS-1 cells (Figure 1D,E). With the overexpression of *TCF2* in β-cells, INS-1 cell viability was restored to control levels (Figure 1F), suggesting *TCF2* to be a crucial link between FFAs and β-cells.

### 2.2. TCF2 Inhibited Reactive Oxygen Species (ROS) Production Stimulated by High Concentrations of Palmitic Acid (PA)

**Figure 2:**
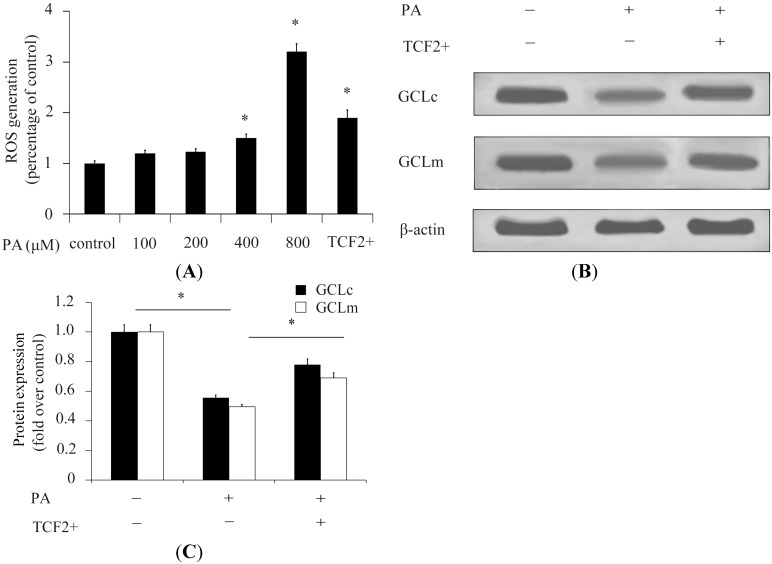
TCF2 inhibits the ROS activation stimulated by high concentrations of PA. ROS generation in INS-1 β-cells was analyzed after treatment with a concentration gradient of PA for 24 h. ROS generation in INS-1 β-cells treated with PA (800 μM) and overexpressing TCF2 was also analyzed (**A**); Expression of GCLc and GCLm was detected by Western blotting (**B**) in INS-1 cells overexpressing TCF2 or not; Levels in cells cultured in DMEM containing 25 mM glucose (control) or 800 μM PA were quantified relative to β-actin (**C**). * *p* < 0.05.

ROS levels are implicated in cell viability, and the high levels of ROS are closely correlated to β-cell apoptosis [18]. We explored whether ROS was implicated in FFA-stimulated decrease of β-cell viability. The results showed that stimulation with high concentrations of PA significantly increased ROS generation compared with control (Figure 2A). With stimulation with 800 μM PA, ROS generation was 3.1-fold greater than that of control (Figure 2A). The overexpression of *TCF2* could significantly decrease ROS generation in β-cells pretreated with 800 μM PA, indicating that TCF2 reduced the toxicity of PA by inhibition of ROS generation (Figure 2A). In addition, the expression of ROS resistance genes (GCLc and GCLm) was evaluated. The results showed that high concentrations of PA significantly decreased GCLc and GCLm expression in β-cells (Figure 2B,C). In contrast, overexpression of *TCF2* could effectively increase GCLc and GCLm expression in β-cells pretreated with high concentrations of PA (Figure 2B,C), suggesting that *TCF2* inhibited PA-induced ROS activation stimulated by increasing expression of ROS resistance genes.

### 2.3. TCF2 Positively Regulated PI3K/AKT and MAPK/ERK Signaling Pathways

**Figure 3:**
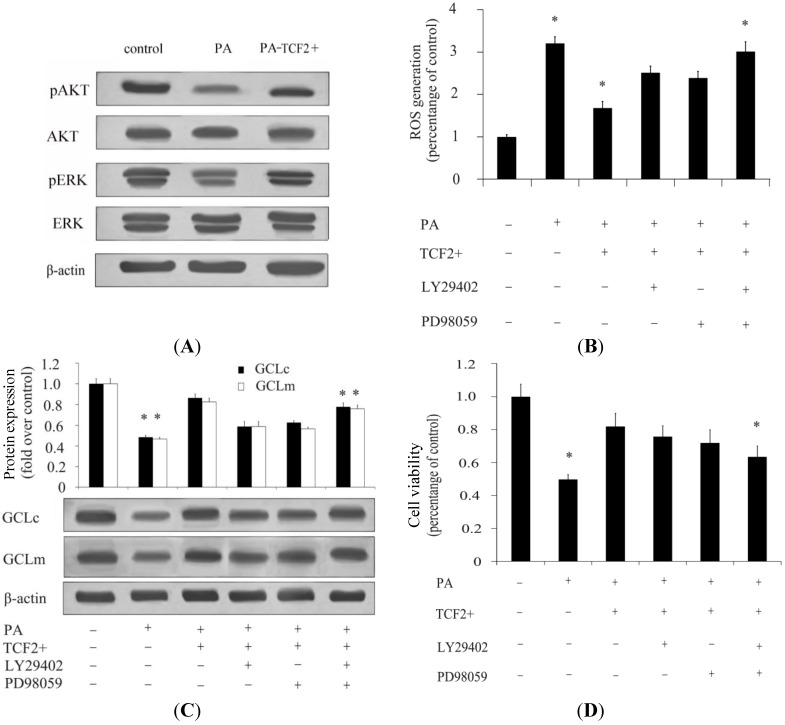
*TCF2* activates PI3K/AKT and MAPK/ERK signaling pathways to inhibit ROS generation in INS-1 cells. INS-1 cells were cultured in DMEM containing 25 mM glucose (control) or 800 μM PA. When needed, 100 nM insulin was added to the culture medium. INS-1 cells overexpressing *TCF2* were cultured in DMEM containing 25 mM glucose and 800 μM PA. After 24 h of incubation, activation of PI3K/AKT and MAPK/ERK signaling pathways was detected by Western blotting (**A**); In the presence of ERK inhibitor PD98059, PI3K inhibitor LY294002, or both inhibitors, ROS generation was detected (**B**); Besides, expression of GCLc and GCLm (**C**) was detected by Western blotting; Cell viability was evaluated (**D**) in the presence of PD98059 and LY294002 in INS-1 cells overexpressing *TCF2* treated with PA and glucose. INS-1 cells cultured in DMEM containing 25 mM glucose or glucose + PA, and INS-1 cells overexpressing *TCF2* cultured in DMEM with glucose + PA were used as controls. * *p* < 0.05.

A previous study has confirmed that PI3K/AKT and MAPK/ERK signaling pathways are closely related to ROS generation in β-cells. Herein, the activation of AKT and ERK was further evaluated. The results showed that high concentrations of PA significantly decreased the activation of AKT and ERK, while overexpression of *TCF2* could restore the activation of AKT and ERK in β-cells pretreated with 800 μM of PA, suggesting that *TCF2* could rescue the substantial inhibition effect on AKT and ERK induced by PA (Figure 3A). After individual inhibition of AKT or ERK using the ERK inhibitor PD98059 or PI3K inhibitor LY294002, ROS generation in β-cells was slightly increased compared with *TCF2*-overexpressed groups (Figure 3B). Simultaneously, more ROS generation was observed when combined with application of the PD98059 and LY294002 (Figure 3B). Thus, these results indicated that PI3K/AKT and MAPK/ERK signaling pathways both play an important role in *TCF2*-mediated modulation of ROS generation. Further analysis of GCLc and GCLm expression showed that *TCF2* overexpression ameliorated the inhibition effect of PA on the expression of GCLc and GCLm. When blocking the AKT and ERK signalings with their sepcific inhibitor significantly attenuated the increase in GCLc and GCLm expression induced by *TCF2* (Figure 3C), implying that *TCF2* decreased ROS generation via PI3K/AKT and MEK/ERK signaling pathways. Besides, the inhibition of PI3K/AKT and MEK/ERK pathways induced a significant decrease of cell viability triggered by *TCF2* (Figure 3D), suggesting a protective effect of *TCF2* on β-cells via regulation of PI3K/AKT and MAPK/ERK signaling to inhibit ROS.

### 2.4. TCF2 Relieved FFA-Abrogated Insulin Secretion

**Figure 4:**
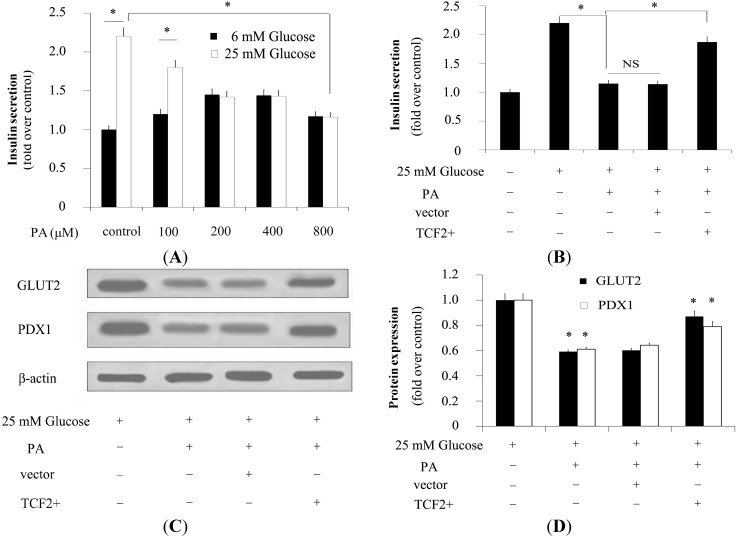
INS-1 cells were cultured in DMEM with 6 mM or 25 mM glucose and pretreated with a concentration gradient of PA to investigate the effect of FFAs on glucose-stimulated insulin secretion (GSIS) (**A**); INS-1 cells pretreated with 6 or 25 mM glucose were cultured in DMEM with PA (800 μM). INS-1 cells with the control vector (blank control) and INS-1 overexpressing *TCF2* were also cultured in DMEM with PA (800 μM) for 24 h. Then insulin secretion was detected to confirm the protective effect of *TCF2* on GSIS (**B**); In addition, the expression of GLUT2 and PDX1 was evaluated by Western blotting (**C**) and quantified (**D**). * *p* < 0.05. NS means no statistically significant.

Other studies suggested that higher concentrations of FFAs significantly inhibited glucose-stimulated insulin secretion (GSIS), even in the presence of high levels of d-glucose [7]. In this study, insulin secretion assay showed that low concentrations of PA (lower than 200 μM) dose dependently stimulated insulin secretion in β-cells pretreated with low levels of glucose (Figure 4A). Conversely, high glucose-stimulated insulin secretion was obviously suppressed by PA treatment (Figure 4A). In addition, overexpression of *TCF2* increased insulin secretion in β-cells, suggesting that it could almost entirely rescue the PA-dependent downregulatory effect of high glucose-stimulated insulin secretion in β-cells (Figure 4B).

### 2.5. TCF2 Positively Modulated Expression of Insulin Secretion-Related Molecules

*GLUT2* and *PDX1.* GLUT2 is in responsible for passive transport of glucose from the blood into cells. PDX1 is a transcriptional regulatory factor of the insulin gene. Both GLUT2 and PDX1 play an important role in regulating insulin secretion. Herein, expression of GLUT2 and PDX1 was analyzed by Western blotting. After treatment with PA, expression of GLUT2 and PDX1 was significantly decreased in β-cells (Figure 4C,D). However, *TCF2* overexpression significantly improved FFA-induced inhibition of GLUT2 and PDX1 in PA-treated β-cells, suggesting that overexpression of *TCF2* could partially restore the inhibition of PA on glucose-stimulated GLUT2 and PDX1 expression. Accordingly, it could be presumed that *TCF2* overexpression could attenuate the inhibitory effect of PA on insulin secrection by regulating GLUT2 and PDX1 expression.

### 2.6. TCF2 Positively Regulated Insulin Secretion via JNK Signaling

**Figure 5:**
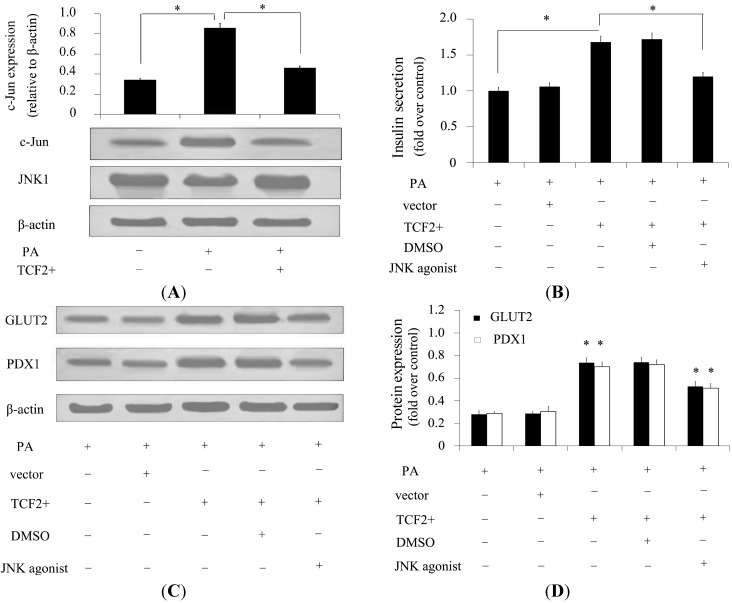
Inhibition of TCF2 decreases insulin secretion via the JNK signaling pathway. INS-1 cells were cultured in DMEM containing 25 mM glucose and pretreated with 800 μM PA to investigate the effect of FFAs on INS-1 gene expression. Activation of JNK signaling was detected by Western blotting (**A**) in INS-1 cells overexpressing *TCF2* or not that were cultured in DMEM containing 25 mM glucose (control) or PA (800 μM). JNK signaling was also detected in INS-1 cells overexpressing *TCF2* cultured in DMEM containing 25 mM glucose and PA (800 μM) (**A**); GSIS was analyzed with DMSO or JNK agonist to confirm the positive regulatory effect of *TCF2* on GSIS through negative regulation of JNK signaling (**B**); In addition, expression of GLUT2 and PDX1 was evaluated by Western blotting (**C**) and quantified (**D**) to evaluate the effect of JNK agonist on gene expression related to insulin secretion. * *p* < 0.05.

JNKs are causally linked to aberrant insulin secretion [19]. Besides, FFAs are reported to be potent JNK activators [20]. However, whether JNKs are associated with *TCF2*-induced insulin secretion remains unclear. To analyze the mechanism underlying the effect of TCF2 on insulin secretion, the activation of c-jun was detected by Western blotting. After treatment with PA, an obvious activation of JNK was detected. However, this increase in JNK activation was dramatically attenuated by *TCF2* overexpression (Figure 5A). Further analysis confirmed that activation of JNK with its specific agonist significantly decreased the insulin secretion induced by *TCF2* overexpression (Figure 5B), implying that *TCF2* may ameliorate FFA-inhibited insulin secretion by suppressing the JNK pathway. To further confirm the role of the JNK signaling pathway in *TCF2*-stimulated insulin secretion, expression of GLUT2 and PDX1 was evaluated. The results showed that, after pretreatment with JNK agonist, TCF2-stimulated overexpression of GLUT2 and PDX1 in β-cells was significantly decreased (Figure 5C,D). Thus, the results suggested that *TCF2* relieved FFA-induced inhibition of insulin secretion via JNK-mediated GLUT2 and PDX1 expression.

## 3. Discussion

A connection between diabetes, obesity, and diet exists in multiple species and is the basis of an escalating human health problem [21]. The β-cell dysfunction and failure are key events in the development of diabetes. Therefore, growth arrest, damage, and death of β-cells are major contributors to the progression of diabetes. A previous study confirmed the close relationship between TCF2 and diabetes. Herein, we showed that expression of *TCF2* was significantly decreased in cultured pancreatic β-cells pretreated with high concentrations of FFAs. Furthermore, overexpression of TCF2 could effectively rescue the decreased viability of β-cells induced by high concentrations of PA, suggesting that *TCF2* plays a crucial role in the function of β-cells.

The mechanisms underlying FFA-induced β-cell apoptosis has drawn increasing attention recently [22]. The evidence shows that FFAs can significantly induce elevation of oxidative stress, resulting in endothelial dysfunction [23]. Besides, oxidative stress is likely involved in the progression of pancreatic β-cell dysfunction and apoptosis, and is thought to be one of the main causes of diabetes [24,25]. To investigate whether ROS is involved in FFA-induced β-cell inviability, we evaluated ROS generation in β-cells. The results showed that high concentrations of PA increased ROS generation, which could be reversed by overexpression of *TCF2* in β-cells, indicating that FFA-induced β-cell inviability was associated with elevation of ROS. Besides, *TCF2* protected β-cells against the toxicity of PA by inhibition of ROS generation. Simultaneously, decreased expression of two ROS inhibition genes, GCLc and GCLm, were up-regulated by overexpression of *TCF2*. Taken together, these results suggest that *TCF2* may ameliorated the the toxicity of PA on β-cell viability by inhibiting ROS pathway.

The accumulating researches have revealed that two major signaling pathways, PI3K/AKT and MEK/ERK pathways, are implicated in β-cell functions and the process of diabetes [26,27]. Recently, data from the literature suggest that Akt and extracellular signal-regulated kinase ERK are involved in regulation of iNOS activity, which is a critical regulator for ROS production [28]. To further clarify the mechanism involved in the inhibitory effect of TCF2 on ROS production, these two signalings were explored. Further analysis indicated that PI3K/AKT and MEK/ERK pathways were inhibited by FFAs. Overexpression of *TCF2* could reverse the suppressive effects of FFA on PI3K/AKT and MEK/ERK, suggesting that *TCF2* can positively modulate PI3K/AKT and MEK/ERK activation. Thus, FFA inhibited cell viability by ROS generation was attenuated. Therefore, these results suggest that PI3K/AKT and MEK/ERK pathways play crucial roles in the regulation effects of TCF2 on ROS and the protective effect in β-cells. However, does *TCF2* activated these pathways directly or indirectly? If it is indirectly, what does interact with *TCF2* during this process? Both of these questions need to be further investigated.

Pancreatic β-cells can produce and secrete multiple glycoprotein factors that, along with insulin, play important roles in normal activity and half-life and directly contribute to glucose homeostasis and peripheral insulin action [2]. The functioning of pancreatic β-cells and normal secretion of insulin are essential for maintaining normal physiological function of the pancreas [29]. Studies have revealed that retention of GSIS exerts a protective role at the onset of diabetes and ranks as a potential aspect against diabetes [30]. Loss of GSIS is a marker of β-cell dysfunction in type 2 DM. Failure of GSIS has been proposed to further provoke the pathogenic onset of type 2 DM in humans [31]. In this study, high concentrations of PA immediately suppressed high GSIS. In contrast, overexpression of *TCF2* led to a significant increase in GSIS in β-cells, suggesting that TCF2 restored the PA-dependent inhibitory effect on GSIS and positively regulated GSIS in β-cells.

A pathogenic tipping point in this pathway may occur when elevated FFA concentrations impair the function of β-cells and reduce insulin secretion. Failure of GSIS has been linked to impaired β-cell GLUT expression. GLUT2, a low-affinity transporter, has been proposed to be active in the control of GSIS [32]. Besides, PDX1, a key regulator of the pancreas, is an important transcription factor required for maintenance of GSIS and β-cell function [33,34]. Further detection of GLUT2 and PDX1 in β-cells confirmed that FFAs led to decreased expression of GLUT2 and PDX1. However, overexpression of TCF2 rescued PA-inhibited GLUT2 and PDX1 expression, confirming that TCF2 positively regulated expression of GLUT2 and PDX1 and contributed to GSIS. JNK signaling interacts with the insulin signaling pathway to influence metabolism, growth, stress tolerance, and regeneration of β-cells [19]. Antagonism between insulin signaling and JNK influences metabolic homeostasis of β-cells [35]. Analysis of the mechanism has confirmed that FFAs activate JNK signaling. In contrast, overexpression of *TCF2* effectively inhibited the activation of JNK and restored GSIS, suggesting that TCF2 ameliorates PA-dependent inhibition of GSIS in β-cells through the suppression of JNK stress signaling.

## 4. Materials and Methods

### 4.1. Cell Culture

Pancreatic INS-1 β-cells were cultured in DMEM (Life Technologies, Gaithersburg, MD, USA) containing 10% FBS (Invitrogen, Grand Island, NY, USA) at 37 °C and 5% CO_2_. Cells in the logarithmic phase of growth were digested using 0.25% trypsin (Genetimes Technology, Shanghai, China) to prepare cell suspensions. The cell suspensions were inoculated onto culture plates and cultured at 37 °C and 5% CO_2_ for 24 h. PA (PerkinElmer, Boston, MA, USA) was dissolved in anhydrous ethanol and diluted to the required concentrations by DMEM with 2% BSA (Calbiochem, La Jolla, CA, USA). When needed, PA solution was added at different concentrations and the cells were further cultured for 48 h.

### 4.2. Cell Viability

Cell viability was analyzed using the MTT (Sigma, St. Louis, MO, USA) method. Cells were firstly seeded onto 96-well plates containing DMEM and cultured at 37 °C for 48 h. After the removal of culture solution, the cells were cultured for a further 48 h after addition of glucose (25 mM) and different concentrations of PA. Then the cells were collected and washed with PBS twice. After addition of 0.5 mg/mL MTT, the cells were cultured at 37 °C and 5% CO_2_ for 4 h. With 150 μL DMSO (Genetimes Technology, Shanghai, China) dissolving, the optical density was detected at 492 nm.

### 4.3. ROS Measurement

ROS were measured using a standard procedure as previously described [36]. In short, cells in 24-well plates were incubated with glucose (25 mM) and/or PA as needed. Then the fluorescent probe 2',7'-dichlorofluorescein diacetate (DCFH-DA; 10 μM, Eastman Kodak, Rochester, NY, USA) was added in the last 30 min of treatment and the cells were incubated in the dark at 37 °C. After washing thrice with PBS, the cells were harvested in PBS with 0.5% Triton X-100 (Sigma, St. Louis, MO, USA) and centrifuged at 12,000× *g* for 15 min. The supernatant was collected. The dichlorofluorescein (DCF) fluorescence intensity of the supernatant was measured at the excitation wavelength of 485 nm and emission wavelength of 530 nm by fluorescence spectrophotometry (Hitachi F-2000; Hitachi Instruments, Tokyo, Japan).

### 4.4. Adenovirus Infection

The TCF2 coding sequence (cds) was used to generate a *TCF2* overexpression vector (It is represented symbolically by *TCF2*+) based on the Shuttle vector (Stratagene, La Jolla, CA, USA). Adenovirus containing the TCF2 cds was constructed by Shanghai JiKaiJi Company. Enhanced GFP (pEGFP) was transfected into INS-1 to allow identification of transfected cells. Briefly, INS-1 cells in the logarithmic phase of growth were inoculated onto 6-well plates (about 5 × 10^4^ cells/well) and cultured at 37 °C and 5% CO_2_. After adding an appropriate amount of adenovirus according to the multiplicity of infection (MOI), the cells were cultured for 3 days. After the addition of puromycin (American Bioanalytical, Natick, MA, USA), they were cultured for 2 days in medium containing INS-1 to obtain cells overexpressing TCF2. An adenovirus producing GFP alone (Ad-GFP) was used as a control. All constructs were confirmed by sequencing.

### 4.5. Insulin Secretion Assay

Insulin secretion measurements of single β-cells were performed at 37 °C in Krebs-Ringer bicarbonate (KRB) buffer containing (in mmol/L): 3.6 KCl, 135 NaCl, 1.5 CaCl_2_, 5 NaHCO_3_, 0.5 NaH_2_PO_4_, 0.5 MgCl_2_, 10 HEPES, and 0.1% BSA (pH 7.4). INS-1 cells of each group were pre-incubated for 1 h in KRB containing 5.5 or 25 mM glucose as required. The supernatants were collected and centrifuged at 13,000× *g* for 2 min to remove residual cells. Samples of 100 μL were assayed for insulin via ELISA (ALPCO, Salem, NH, USA). Results were standardized by total protein content and are expressed as insulin released as a percentage of the total insulin content.

### 4.6. Real-Time Reverse Transcription Polymerase Chain Reaction (RT-PCR).

Total RNA was prepared using the Trizol reagent (Invitrogen, Carlsbad, CA, USA) and the ratio of optical absorbance ratio at 260 and 280 nm (A260 nm/280 nm) was measured to determine the content. Then, the RNA was reverse-transcribed with random hexamer primers to obtain cDNA. Quantitative RT-PCR analysis was performed with cDNA as the template to amplify *TCF2* mRNA with specific primers (Table 1). Quantitative RT-PCR with SYBR green detection was performed using specific primers. *TCF2* mRNA was normalized to mouse β-actin mRNA with the comparative *C*t method.

### 4.7. Western Blotting

Cells were lysed with 200 μL lysis buffer containing 20 mmol/L HEPES, 25 mmol/L MgCl, 5 mmol/L KCL, 0.5% (*v*/*v*) complete protease inhibitor, and Triton X-100. Then, the debris was removed by centrifugation at 12,000× *g* at 4 °C for 10 min. Equal amounts of cell protein (typically 80 µg) were separated using 8% precast SDS-PAGE gels (Invitrogen, Carlsbad, CA, USA) and electrophoretically transferred to PVDF membranes (Millipore, Beijing, China). The membranes were subsequently probed individually with the following polyclonal primary antibodies: TCF2 antibody (1:500, ProSci Inc., Poway, CA, USA); GLUT2 antibody, PDX1 antibody, GCLc antibody, GCLm antibody, pAKT antibody, AKT antibody, pERK antibody, and ERK antibody (1:500, BD Transduction Laboratories, San Jose, CA, USA). Detection was performed by incubation with goat anti-mouse immunoglobulin G (IgG; 1:5000, Sigma, St. Louis, MO, USA) followed by enhanced chemiluminescence (ECL, Amersham Pharmacia Biotech, Ltd., Piscataway, NJ, USA). The intensity of the bands was measured using an image analysis system with Image-Pro Plus 6.0 software (Media Cybernetics, Silver Springs, MD, USA).

### 4.8. Statistical Analysis

All the data obtained were analyzed using spss 16.0 statistical software. The results are expressed as mean ± standard deviation (SD). All experiments were performed independently at least three times. Differences between two groups were analyzed by Student’s *t*-test. Differences between multiple groups were analyzed by ANOVA. *p* < 0.05 was considered statistically significant.

## 5. Conclusions

Our work has uncovered a potential link between *TCF2* and the effects of high concentrations of FFAs on pancreatic β-cells. In particular, we found that TCF2 had a protective effect on FFA-induced cytotoxicity in pancreatic β-cells by negatively modulating ROS generation, via the PI3K/AKT and MEK/ERK signaling pathways. Furthermore, a protective effect of *TCF2* on GSIS by positive modulation of JNK-PDX1 and GLUT2 signaling was also confirmed. Taken together, our study confirmed that overexpression of *TCF2* could protect β-cells against the oxidative damage and inhibition of GSIS induced by high concentrations of PA. Hence, this study suggests that *TCF2* may be a new therapeutic target to achieve prevention and treatment of diabetes.
